# Decreased Cerebrospinal Fluid Orexin-A (Hypocretin-1) Concentrations in Patients after Generalized Convulsive Status Epilepticus

**DOI:** 10.3390/jcm9103354

**Published:** 2020-10-19

**Authors:** Mojdeh Samzadeh, Ewa Papuć, Marzena Furtak-Niczyporuk, Konrad Rejdak

**Affiliations:** 1 Department of Neurology, Medical University of Lublin, 20-954 Lublin, Poland; formojdeh@aol.com (M.S.); ewapap@yahoo.pl (E.P.); 2 Department of Public Health, Medical University of Lublin, 20-954 Lublin, Poland; marzenafurtakniczyporuk@umlub.pl

**Keywords:** biomarker, generalized convulsive status epilepticus, hypocretin 1, orexin-A, cerebrospinal fluid

## Abstract

The effects of status epilepticus on the orexin/hypocretin system have yet to be investigated. The present study aimed to assay orexin-A/hypocretin-1 in the cerebrospinal fluid (CSF) of patients after generalized convulsive status epilepticus (GCSE). The study groups included 20 GCSE patients, 24 patients diagnosed with epilepsy but remaining in remission (ER), and 25 normal controls (CTR). Diagnostic lumbar puncture was performed in GCSE patients within 3–10 days of seizure cessation, as well as in the ER and to CTR subjects. Among all GCSE patients, the outcome was graded according to the modified Rankin Scale (mRS) at 1-month follow-up. Orexin-A levels were measured in unextracted CSF samples, using a commercial radioimmunoassay. There was a significant overall difference in median CSF orexin-A concentrations between GCSE, RE, and CTR patients (*p* < 0.001). The lowest concentrations were noted in the GCSE group compared to ER (*p* < 0.001) or CTR (*p* < 0.001). CSF orexin-A levels in GCSE patients inversely correlated with clinical outcome as assessed on the mRS at 1-month follow-up (*r* = −0.55; *p* = 0.1). In conclusion, CSF orexin-A levels may serve as a biomarker of increased turn-over of the peptide or post-SE neuronal damage, and implicates the orexin system in the pathogenesis of SE.

## 1. Introduction

Generalized convulsive status epilepticus is a medical emergency with a complex pathophysiology. A number of systemic changes related to ongoing seizure activity have been identified in detail, while the molecular scenario in the central nervous system is only partly understood. The fundamental principle involves the failure of endogenous mechanisms to terminate a seizure. This failure can occur because of excessive abnormal excitation or from a loss of endogenous inhibitory mechanisms. These maladaptive changes allow a single seizure to transform into status epilepticus and contribute to the self-perpetuating nature and pharmaco-resistance of the disorder [1].

The orexin system (also known as the hypocretin system) has received a great deal of attention due to its multiple physiological activities, mainly related to the control of the wake/sleep cycle [2]. There is also accumulating evidence that it can be very important in the context of epilepsy [3]. Experimental studies have reported pro-convulsant activity of orexin and other orexin receptor agonists [4,5], while antagonists were potent inhibitors of seizures in different rodent models [6,7,8]. Previously, we reported lowered concentrations of orexin-A cerebrospinal fluid in patients suffering from repetitive generalized seizures [9]. This finding might suggest that orexin-A could serve as a biomarker in patients suffering from uncontrolled prolonged seizures.

The current study focused on orexin-A concentrations in patients with generalized convulsive status epilepticus in relation to clinical outcomes, in order to shed more light on the complex pathophysiology of status epilepticus (SE) and the orexin system involvement.

## 2. Materials and Methods

### 2.1. Patients

The study protocol was approved by the local ethics committee. This was a prospective study conducted at the Department of Neurology of the Medical University of Lublin, which is a tertiary center for a region with a population of about 2.1 million. Informed consent for participation in the study was obtained from each subject (or from the next of kin if the patient was incapable).

The study groups included patients suffering from generalized tonic-clonic status epilepticus, patients diagnosed with epilepsy but remaining in remission, and normal controls.

SE was defined according to the ILAE proposal as a condition resulting either from the failure of the mechanisms responsible for seizure termination or from the initiation of mechanisms that lead to abnormally prolonged seizures (after time point t 1 = 5 min). It is a condition that can have long-term consequences (after time point t 2 = 30 min), including neuronal death, neuronal injury, and alteration of neuronal networks, depending on the type and duration of seizures [10].

On admission to the emergency ward, the patients were subjected to a standard diagnostic workup, including neurological examination, blood biochemical assessment, and CT scan. Subsequently, the patients were admitted to a hospital neurological ward for further evaluation.

Treatments: Each SE patient was treated in accordance with our internal protocols according to national guidelines for SE [11]. Intravenous diazepam was administered as a first-line antiepileptic drug (AED) with a dose not exceeding 30 mg (range 5–30 mg). In non-responders, the patients were admitted to NICU (Neurological Intensive Care Unit) and were treated with second-line intravenous valproic acid or phenytoin followed by anesthetic drugs—a third-line option in cases of refractoriness. All SE patients had intensive monitoring, including laboratory assessment and EEG (electroencephalographic) recordings. In addition, cerebrospinal fluid (CSF) examination was part of the diagnostic workup in order to exclude infectious or inflammatory causes of SE, and it was performed after seizure cessation.

Outcome assessment: The severity of the disease and its consequences were assessed using the modified Rankin Scale (mRS) [12]. On the day of discharge, all patients underwent a neurologic examination performed by a neurologist, and the outcome was graded according to the modified Rankin Scale. In those who survived, a good outcome was defined as an mRS score 0–2 and a poor outcome as an mRS score 3–5, while a score of 6 was assigned to those who died.

In addition, patients diagnosed with epilepsy but remaining in remission (ER) (defined as the absence of seizures for at least 3 months prior to inclusion in the study), who were admitted in order to clarify their disease status, were included. The control group consisted of subjects without any diagnosed organic neurological disease.

Patients had brain imaging with MRI and lumbar puncture as standard diagnostic evaluation techniques.

### 2.2. Biochemical Analysis

Lumbar puncture was performed within 48 h of generalized tonic-clonic seizure (GTCS) cessation (range: 5–48 h) in SE patients. For both patients and controls, CSF sampling was performed between the hours of 7 a.m. and 5 p.m., and the samples were immediately frozen and stored at −80 °C until analysis.

Orexin-A levels were measured in unextracted samples, using a standardized radioimmunoassay (Phoenix Pharmaceuticals, Inc., Phoenix Europe GmbH, Karlsruhe, Germany) with a detection limit of 100 pg/mL as described previously [13]. Investigators conducting orexin-A assays were blinded for clinical information on the patients.

### 2.3. Statistical Analysis

Comparisons between unpaired non-parametric groups were made using non-parametric tests (Kruskal–Wallis ANOVA and Mann–Whitney U-test for the post-hoc testing). The linear relation between parameters was assessed by the Spearman rank correlation coefficient (r). The calculations were performed with GraphPad InStat 3.05 software (GraphPad Software, Inc. San Diego, CA, USA). The data for orexin-A concentrations are presented as medians and range throughout the manuscript.

## 3. Results

### 3.1. Demographics and Clinical Characteristics

We recruited 20 patients with generalized convulsive status epilepticus (GCSE) and 24 with epilepsy in remission. The control group consisted of 10 subjects experiencing non-specific clinical symptoms, and 15 patients with long-lasting headaches, which at discharge after exclusion of other causes were finally classified as tension-type headaches.

The demographic and clinical characteristics of the study groups are presented in Table 1 and Table 2. There were no significant differences with regard to age and gender between the subjects from the three groups.

Among the epilepsy patients in remission (*n* = 24), all had focal onset seizures in their histories (15 from temporal and 9 from frontal lobe), and all received treatment with stable doses for the last 3 months prior to admission (19 on monotherapy and 5 on duotherapy). The majority of the patients had unknown etiology of epilepsy, while only four had remote structural cortical damage of ischemic origin. In the GCSE group, there were 13 patients with a previous diagnosis of epilepsy (7 with remote structural etiology) who stopped treatment and 6 with unknown etiology. Table 2 presents further characteristics of the patients with GCSE. Seven patients with GCSE had new onset of SE of unknown etiology. The median duration of SE was 8 h [range 1–9], and the seizures were stopped by the escalation of treatment, with 25% of patients requiring intravenous anesthetics. Fifteen patients (75%) returned to normal independent functioning at discharge, while five patients had poor outcome as assessed at the 3-month follow-up. Three patients did not regain consciousness despite seizure cessation on EEG monitoring, of whom one patient died and two patients had severe disability with cognitive and behavioral deficits.

### 3.2. Biochemical and Clinical Outcomes

**Figure 1:**
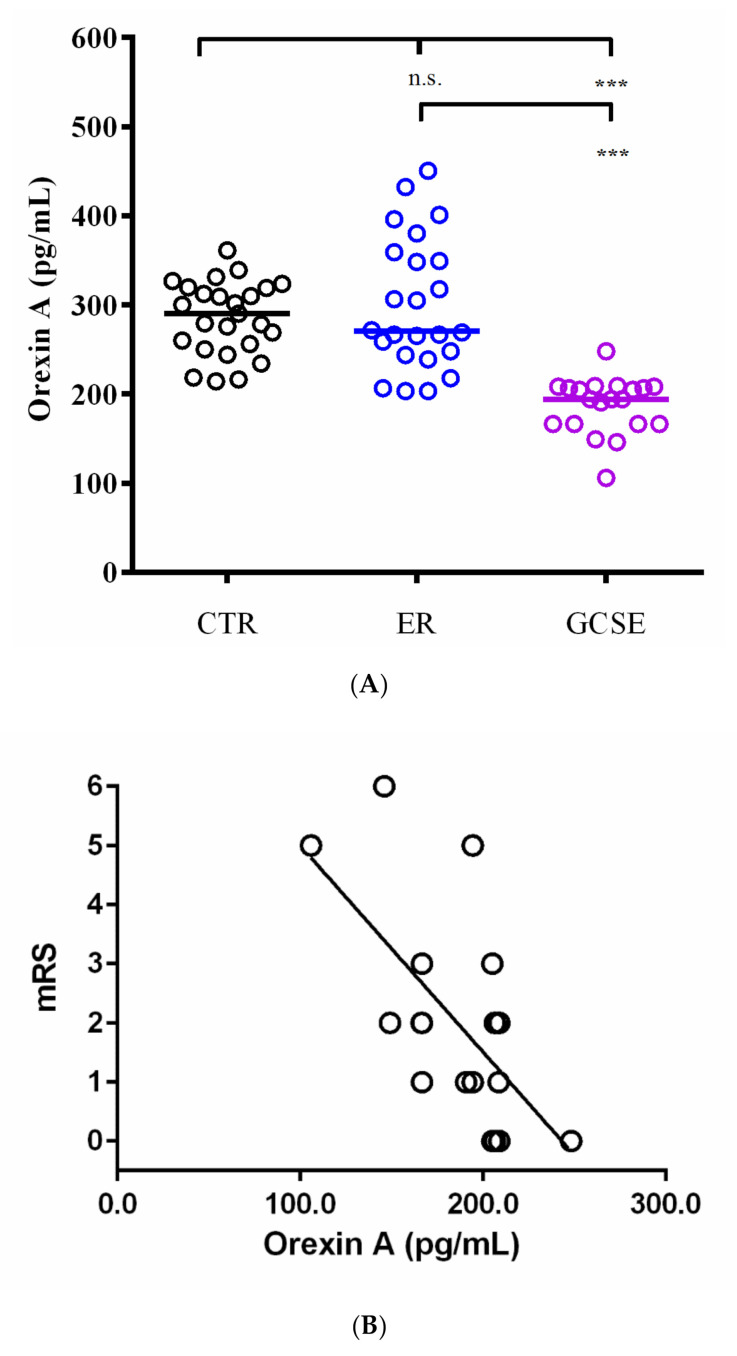
(**A**) Cerebrospinal fluid (CSF) orexin-A levels (pg/mL) in controls (CTR), epilepsy patients in remission (ER), and patients with generalized convulsive status epilepticus (GCSE) (GCSE vs. CTR, *p* < 0.001; GCSE vs. ER, *p* < 0.001; ER vs. CRT, *p* > 0.05); *** *p* < 0.001; n.s. = not significant. (**B**) Cerebrospinal fluid (CSF) orexin-A levels (pg/mL) correlated with modified Rankin Score (mRS) in patients with GCSE (Spearman, *r* = −0.55; *p* = 0.01).

Basic CSF examination revealed no significant pathological changes in any study subjects. There was a significant, overall difference in median CSF orexin-A concentrations between controls (314.1 pg/mL [234.4–379.1]), epileptic patients in remission (305.6 pg/mL [203.4–450.5]), and GCSE patients (194.3 pg/mL [105.8–248.1]; *p* < 0.001). Then, post-hoc comparisons between groups were performed. As presented in Figure 1, the lowest levels were in the GCSE patients as compared with ER (*p* < 0.001) and control patients (*p* < 0.001). There was no statistically significant difference between the ER and control groups (*p* > 0.05).

Eleven patients out of twenty (55%) in the GCSE group had orexin-A levels below the cut-off value of 200 pg/mL, which represents intermediate, low narcolepsy concentrations, previously established using the same assay [13].

There was no significant correlation between CSF orexin-A concentration and the time elapsed between seizure cessation and the lumbar puncture (*p* > 0.05) in the GCSE group. The duration of SE tended to inversely correlate with the orexin-A levels as measured after SE cessation (*r* = −0.42; *p* = 0.06). CSF orexin-A levels in GCSE patients were inversely correlated with clinical outcomes as assessed on mRS at 1-month follow-up (Spearman *r* = −0.55; *p* = 0.01).

## 4. Discussion

This was the first study to assess the CSF concentrations of orexin-A in patients suffering from GCSE. The results are consistent with our previous report on patients with repetitive seizures [9]. Now, we demonstrate that prolonged seizures may lead to lowered concentrations of orexin-A in cerebrospinal fluid as assessed 3–10 days after the seizure cessation. Interestingly, lower CSF orexin-A levels tended to inversely correlate with the duration of status epilepticus. Moreover, there was a negative correlation with clinical outcome measure, meaning that the lower the CSF orexin-A level post-SE, the worse the prognosis for the patient during the 1-month observation period. These results suggest that CSF orexin-A might be a useful prognostic biomarker for SE patients.

Indeed, there is accumulating evidence that the orexin system might be involved in the pathogenesis of epilepsy and status epilepticus. Early experimental studies demonstrated that orexins are excitatory peptides and regulate the wake-sleep cycle and other behavioral functions [14,15]. There was also a hint that orexins might be involved in epileptogenesis, since the application of OX-A (Orexin-A) to hippocampal slices modulated the balance between GABA-ergic (gamma-Aminobutyric acid) and glutamatergic neurotransmissions [16].

Another study has investigated a variation in orexin expression in the brains of rats after pilocarpine-induced status epilepticus (Pilo-SE) [17]. Interestingly, the expression of prepro-OX mRNA was found in the hippocampus, although it was 50-fold lower compared to the hypothalamus. The prepro-OX mRNA levels dramatically decreased in the hypothalamus, with the minimum being observed on day 1 with subsequent normalization on day 3 post-SE. The density of both OX-A and OX-B fibers in the hypothalamus decreased significantly at day 2 post-SE vs. controls, where labeling appeared to be disorganized. It culminated at day 4 post-SE, suggesting that alterations in the hypocretin-1 and hypocretin-2 systems may last for a long period of time following severe GTCS. However, the authors observed increased expression of orexin-B in the hippocampus within both neurons and astrocytes, which they associated with the process of SE-evoked epileptogenesis.

Another study evaluated the influence of SB 334867 (a selective OX1 receptor antagonist) and EMPA (a selective OX2 receptor antagonist) on seizure thresholds in mice [7]. The authors also determined the changes in the orexin-A level following different types of seizures. It was shown that blocking OX1 receptors suppressed seizures while concentrations of orexin-A were increased in homogenized brain tissue as measured immediately after SE. However, this provides a rather crude estimation of dynamic changes in neuropeptide concentrations during and after prolonged seizures. It remains to be studied whether orexin-A might initiate spontaneous seizures or if its increase in the brain tissue is a rather secondary event.

The apparent weakness of our study is that we were unable to assess the effects of different antiepileptic drugs on CSF orexin-A concentrations in our patients. Indeed, it is a potential confounding factor, especially considering benzodiazepines and barbiturates. However, based on the limited available evidence, there is no clear interaction between benzodiazepines and barbiturates and the orexin system [18,19].

## 5. Conclusions

We report decreased orexin-A levels in CSF in 3–10 days after spontaneous prolonged SE in human subjects. The findings could indicate CSF orexin-A levels may serve as a biomarker of increased turnover of the peptide and depletion from the hypothalamus, which could be implicated in the pathogenesis of SE. Orexin-A concentrations inversely correlated with clinical outcomes after SE, which seems to indicate that post-SE neuronal damage could be an important cause in this respect since it happens after acute brain injury [20,21].

## Figures and Tables

**Table 1:** Study groups characteristics.

Characteristics	CTR	ER	GCSE	*p*-Value
*n*	25	24	20	NA
Age (median, range; years)	43 (25–70)	41 (26–73)	46 (23–71)	n.s.
Gender (F/M)	13–12 month	14–10 month	11–9 month	n.s.
Epilepsy diagnosis before admission (*n*, %)	0 (0%)	24 (100%)	13 (65%)	NA
Duration since epilepsy diagnosis (median, range; years)	*n*/A	10 (2–15)	5 (0–14])	NA
Etiology (n, %)				
-unknown	*n*/A	20 (83%)	6 (46%)	NA
-structural	*n*/A	4 (17%)	7 (54%)	
-other	*n*/A	0 (0%)	0 (0%)	

Abbreviations: *n* = no. of patients; CTR = controls; ER = epilepsy in remission; GCSE = generalized convulsive status epilepticus; F = female; M = male; n.s = not significant.

**Table 2:** Status epilepticus patients’ characteristics.

Characteristics	GCSE Group
*n*	20
SE duration (median, range, hours)	8 (1–72)
Time elapsed between CSF collection and SE cessation (days, median, range)	3 (1–9)
Treatments for GCSE	
-benzodiazepines	20 (100%)
-intravenous valproic acid	13 (90%)
-intravenous phenytoin	5 (25%)
-intravenous anesthetics	5 (25%)
Outcome at 3-month follow-up	
-good outcome (0–2 mRS)	15 (75%)
-poor outcome (3–5 mRS)	4 (20%)
-death (6 mRS)	1 (5%)

Abbreviations: *n* = no. of patients; GCSE = generalized convulsive status epilepticus; CSF = cerebrospinal fluid; mRS = modified Rankin Scale.

## References

[1] Betjemann J.P., Lowenstein D.H. (2015). Status epilepticus in adults. Lancet Neurol..

[2] Kornum B.R., Faraco J., Mignot E. (2011). Narcolepsy with hypocretin/orexin deficiency, infections and autoimmunity of the brain. Curr. Opin. Neurobiol..

[3] Ng M.C. (2017). Orexin and Epilepsy: Potential Role of REM Sleep. Sleep.

[4] Kortunay S., Erken H.A., Erken G., Genç O., Sahiner M., Turgut S., Turgut G. (2012). Orexins increase penicillin-induced epileptic activity. Peptides.

[5] Ni L.Y., Zhu M.J., Song Y., Liu X.M., Tang J.Y. (2014). Pentylenetetrazol-induced seizures are exacerbated by sleep deprivation through orexin receptor-mediated hippocampal cell proliferation. Neurol. Sci..

[6] Goudarzi E., Elahdadi-Salmani M., Lashkarbolouki T., Goudarzi I. (2015). Hippocampal orexin receptors inactivation reduces PTZ induced seizures of male rats. Pharmacol. Biochem. Behav..

[7] Socała K., Szuster-Ciesielska A., Wlaź P. (2016). SB 334867, a selective orexin receptor type 1 antagonist, elevates seizure threshold in mice. Life Sci..

[8] Asadi S., Roohbakhsh A., Shamsizadeh A., Fereidoni M., Kordijaz E., Moghimi A. (2018). The effect of intracerebroventricular administration of orexin receptor type 2 antagonist on pentylenetetrazol-induced kindled seizures and anxiety in rats. BMC Neurosci..

[9] Rejdak K., Papuć E., Grieb P., Stelmasiak Z. (2009). Decreased cerebrospinal fluid hypocretin-1 (orexin A) in patients after repetitive generalized tonic-clonic seizures. Epilepsia.

[10] Trinka E., Cock H., Hesdorffer D., Rossetti A.O., Scheffer I.E., Shinnar S., Shorvon S., Lowenstein D.H. (2015). A definition and classification of status epilepticus—Report of the ILAE Task Force on Classification of Status Epilepticus. Epilepsia.

[11] Jędrzejczak J., Mazurkiewicz-Bełdzińska M., Szmuda M., Majkowska-Zwolińska B., Steinborn B., Ryglewicz D., Owczuk R., Bartkowska-Śniatkowska A., Widera E., Rejdak K. (2018). Convulsive status epilepticus management in adults and children: Report of the Working Group of the Polish Society of Epileptology. Neurol. Neurochir. Pol..

[12] Bonita R., Beaglehole R. (1988). Modification of Rankin Scale: Recovery of motor function after stroke. Stroke.

[13] Mignot E., Lammers G.J., Ripley B., Okun M., Nevsimalova S., Overeem S., Vankova J., Black J., Harsh J., Bassetti C. (2002). The role of cerebrospinal fluid hypocretin measurement in the diagnosis of narcolepsy and other hypersomnias. Arch. Neurol..

[14] Hagan J.J., Leslie R.A., Patel S., Evans M.L., Wattam T.A., Holmes S., Benham C.D., Taylor S.G., Routledge C., Hemmati P. (1999). Orexin A activates locus coeruleus cell firing and increases arousal in the rat. Proc. Natl. Acad. Sci. USA.

[15] Ida T., Nakahara K., Katayama T., Murakami N., Nakazato M. (1999). Effect of lateral cerebroventricular injection of the appetite-stimulating neuropeptide, orexin and neuropeptide Y, on the various behavioral activities of rats. Brain Res..

[16] Selbach O., Doreulee N., Bohla C., Eriksson K.S., Sergeeva O.A., Poelchen W., Brown R.E., Haas H.L. (2004). Orexins/hypocretins cause sharp wave- and theta-related synaptic plasticity in the hippocampus via glutamatergic, gabaergic, noradrenergic, and cholinergic signaling. Neuroscience.

[17] Morales A., Bonnet C., Bourgoin N., Touvier T., Nadam J., Laglaine A., Navarro F., Moulin C., Georges B., Pequignot J.M. (2006). Unexpected expression of orexin-B in basal conditions and increased levels in the adult rat hippocampus during pilocarpine-induced epileptogenesis. Brain Res..

[18] Kushikata T., Hirota K., Yoshida H., Kudo M., Lambert D.G., Smart D., Jerman J.C., Matsuki A. (2003). Orexinergic neurons and barbiturate anesthesia. Neuroscience.

[19] He Y., Kudo M., Kudo T., Kushikata T., Li E., Hirota K. (2007). The effects of benzodiazepines on orexinergic systems in rat cerebrocortical slices. Anesth. Analg..

[20] Ripley B., Overeem S., Fujiki N., Nevsimalova S., Uchino M., Yesavage J., Di Monte D., Dohi K., Melberg A., Lammers G.J. (2001). CSF hypocretin/orexin levels in narcolepsy and other neurological conditions. Neurology.

[21] Rejdak K., Petzold A., Lin L., Smith M., Kitchen N., Thompson E.J. (2005). Decreased CSF hypocretin-1 (orexin-A) after acute haemorrhagic brain injury. J. Neurol. Neurosurg..

